# Prevalence of psychopathy in a community sample of Spanish adults: Definitions and measurements matter

**DOI:** 10.3389/fpsyg.2022.997303

**Published:** 2022-10-28

**Authors:** Ana Sanz-García, María Elena Peña Fernández, María Paz García-Vera, Jesús Sanz

**Affiliations:** Department of Personality, Assessment, and Clinical Psychology, Complutense University of Madrid, Madrid, Spain

**Keywords:** psychopathy, prevalence, general population, personality traits, five-factor model, Big Five model

## Abstract

The main objective of this work is to examine the prevalence of psychopathy in the general adult population from the main currently existing theoretical perspectives of psychopathy, using for this purpose the five-factor or Big Five model as a common language that allows the comparison and integration of the personality traits considered as defining psychopathy by these different perspectives. The NEO Personality Inventory-Revised (NEO PI-R) was applied to a sample of 682 adults of the general Spanish population. The prevalence of clinical and subclinical psychopathy was calculated according to six different definitions of these two constructs based on Hare’s, Lilienfeld’s, triarchic, and DSM-5-hybrid models, and the simultaneous presence of a minimum number of personality traits that differed from the sample mean by one standard deviation. Prevalence rates for the different definitions were consistently low, indicating that the prevalence of clinical psychopathy in the general Spanish population is around 0.55%, and that of subclinical psychopathy is around 1.65%. There were no significant sex differences in the prevalence of psychopathy. These results question the alarmist claims that warn about the existence in society of a very high number of people with psychopathy who can cause many social, economic, physical, and psychological damage to others.

## Introduction

Psychopathy is a multidimensional personality and clinical construct marked in part by antisocial behaviors and an increased likelihood for committing crimes (Hare, 1993, 2003; Crego and Widiger, 2018). A growing scientific literature conceives psychopathy as a maladaptive variant of the normal personality. From this dimensional perspective, there is strong interest in examining the presence and influence of psychopathy in everyday life (Dutton, 2012; Babiak and Hare, 2019; Fritzon et al., 2020). In this sense, some scientific publications have warned that a significant percentage of the general population has psychopathy, a percentage that in some studies and in some subpopulations may reach 6% (Hagnell et al., 1994) or even 12% (Love and Holder, 2014) or 21% (Fritzon et al., 2017). The existence of such a high number of people with psychopathy may be surprising and even disturbing, given the social, economic, physical, and psychological damage that psychopaths supposedly produce in many people around them (Hare, 1993; Babiak and Hare, 2019).

Recently, Sanz-García et al. (2021) conducted a meta-analysis of studies examining the prevalence of psychopathy in the general population. The results of this meta-analysis suggest that: (1) there are only 15 published studies on this topic; (2) the averaged prevalence of psychopathy is 4.5% obtained from samples of university students, workers from different organizations, and adults of the community from the USA, the United Kingdom, Canada, Australia, Sweden, Portugal, and Belgium; (3) compared to women, men have significantly higher prevalence rates of psychopathy; and (4) prevalence rates show much variation as a function of instrument type for measuring psychopathy; thus, compared to self-report instruments of psychopathic personality traits such as, for example, the Psychopathic Personality Inventory-Revised (PPI-R; Lilienfeld and Widows, 2005), lower prevalence rates of psychopathy (1.2%) are obtained if clinical rating instruments such as the Hare Psychopathy Checklist-Revised (PCL-R; Hare, 2003) are used for measuring psychopathy.

### Models and definitions of psychopathy

The differences found in the prevalence rates of psychopathy in the general adult population as a function of instrument type arise from the different theoretical approaches in defining psychopathy that characterize its research. The PCL-R, for example, is based on a model of psychopathy that includes most of the affective, interpersonal, and social personality characteristics that Cleckley (1976) proposed in his classic clinical definition of psychopathy. According to Cleckley, psychopathy comprises personality traits related to a lack of emotion (e.g., absence of remorse or feelings of guilt, superficial affection, affective insensitivity, absence of empathy) and the presence of ethically reprehensible interpersonal behaviors (e.g., falsehood and insincerity, manipulation) and socially maladapted behavior (e.g., parasitic lifestyle, poor self-control of behavior, promiscuous sexual behavior, the absence of realistic long-term goals, inability to accept responsibility for one’s actions), but also related to some behaviors that give an outward appearance of normalcy (e.g., superficial charm, ease of speech). However, the model of psychopathy that underlies the PCL-R, that is, the Hare’s (2003) model of psychopathy, gives more weight to the socially deviant lifestyle and characteristics of criminality than the original conception of Cleckley. In fact, based on factor analysis results, Hare (2003) suggests that 18 of the 20 PCL-R items form four correlated factors, including Antisocial and Lifestyle factors along with Interpersonal and Affective factors.

Nowadays, along with the Hare’s model and its principal measurement, the PCL-R, there are at least other three main models of psychopathy with their corresponding measurements: that of Lilienfeld (Lilienfeld and Widows, 2005), operationalized by the PPI-R, that of the triarchic model of Patrick et al. (2009), operationalized by the Triarchic Psychopathy Measure or TriPM (Patrick, 2010), and that of the DSM-5’s hybrid model of personality disorders (American Psychiatric Association, 2013), operationalized by the Personality Inventory for DSM-5 (PID-5; Krueger et al., 2012).

Both the Lilienfeld’s and triarchic models exclude from their psychopathy definitions the presence of criminal or even antisocial or unethical behaviors, which are considered more as a possible consequence of psychopathy and not so much as a central component of it. These two models also include as essential elements of psychopathy not only maladaptive personality traits but also some clearly adaptive traits related to positive mental health. For example, the model of psychopathy of Lilienfeld underlying the PPI-R implies the existence of two higher-order factors or dimensions, each of which groups together several of those personality traits (Lilienfeld and Widows, 2005). The first factor, called Fearless Dominance, groups three personality traits: social influence, fearlessness, and stress immunity. This is a factor that includes clearly adaptive personality characteristics. The second factor, called Self-Centered Impulsivity, groups other four personality traits: Machiavellian egocentricity, impulsive non-conformity, blame externalization, and carefree non planfulness. The model also proposes an eighth personality trait, coldheartedness, which does not load on either factor or show any relationship with them and represents in itself a third dimension of psychopathy.

The proposal of a dimension or factor of Fearless Dominance in the PPI-R influenced the development of the triarchic model of psychopathy of Patrick et al. (2009). This model proposes that the following three personality constructs are essential to understanding psychopathy: Disinhibition, Meanness, and Boldness, which can be measured through the TriPM (Patrick, 2010). These three personality dimensions are conceived with greater amplitude than personality traits in other instruments and models and are more similar to the dimensions, factors, or subfactors that these other instruments measure. In fact, the triarchic model of psychopathy was developed as a framework that allows the integration of personality traits with neurobiological and behavior indices (e.g., indices of event-related potential reactivity, startle potential, and facial processing).

A different theoretical perspective on psychopathy is that of the alternative hybrid model of classification of personality disorders of the DSM-5 (American Psychiatric Association, 2013; Crego and Widiger, 2018). This model proposes that psychopathy is a variant of antisocial personality disorder and that the diagnosis of antisocial personality disorder should be based in the presence of six or more of the following seven pathological personality traits: manipulativeness, deceitfulness, callousness, hostility, irresponsibility, impulsivity, and risk-taking. In addition, the DSM-5 proposes that the psychopathic variant of the antisocial personality disorder is characterized by low levels of anxiousness and withdrawal, and high levels of attention seeking (American Psychiatric Association, 2013). The ten personality traits that define the psychopathy according to the DSM-5 can be measured through the PID-5.

Since there are different perspectives or definitions of psychopathy, the development of a valid and consensual definition is one of the main challenges facing research on this area. In the meantime, however, it seems that it would be best to examine the various issues of psychopathy, such as its prevalence in the general adult population, from all these perspectives and to examine and take into consideration the results that show high consistency among the different perspectives.

### Psychopathy and the Big Five model

To examine the prevalence of psychopathy from the different perspectives on psychopathy, it is useful to have a common language that allows the comparison and integration of personality traits that the different perspectives consider as defining psychopathy. This common language can be provided by the five-factor or Big Five model of personality, which, in the last 30 years, has become the most consensual and validated taxonomy of personality traits (McCrae and Costa, 2003). The Big Five model proposes that five global dimensions of personality called Neuroticism, Extraversion, Openness to Experience, Agreeableness, and Conscientiousness can summarize and integrate most of the relevant personality traits. The NEO Personality Inventory-Revised (NEO PI-R; Costa and McCrae, 1992) is one of the first instrument specifically developed to operationalize the five-factor model and has become the standard for the evaluation of those five global dimensions. The NEO PI-R also measures 30 specific personality traits or facets, six per dimension.

A good number of studies have examined the relationships of psychopathy with the facets of the five-factor model measured by the NEO PI-R. The results of these studies have been summarized in the meta-analyses of Decuyper et al. (2009) and O’Boyle et al. (2015), and in the study of Lynam and Miller (2015). The mean correlations obtained in these three works are, in general, quite consistent and, taking into account the correlations that were statistically significant in the three works (see Supplementary Table 1A), it can be concluded that psychopathy is related to 13 facets of the five-factor model, of which six belong to Agreeableness (trust, straightforwardness, altruism, compliance, modesty, and tender-mindedness), three to Conscientiousness (dutifulness, self-discipline, and deliberation), two to Neuroticism (angry hostility and impulsivity), and two to Extraversion (warmth and excitement-seeking).

Moreover, a good number of studies have identified consistent and theoretically meaningful associations between the personality traits of the five-factor model and the personality traits of psychopathy both regarding the most classic proposals of psychopathy such as, for example, that operationalized by Hare’s PCL-R, and regarding the most current proposals such as, for example, those operationalized by the PPI-R, the triarchic model of psychopathy, or the hybrid model of personality disorders of the DSM-5. For example, Widiger and Lynam (1998) have proposed the correspondence between the personality traits of psychopathy measured by the PCL-R and the facets of the NEO PI-R based on the item-by-item content analysis of the PCL-R and its translation in terms of the facets of the NEO PI-R. Many of these facets also coincide with the prototype of a person with psychopathy, according to the average rating of 21 researchers in psychopathy who were asked to “rate the prototypical psychopath” using 30 bipolar scales that corresponded to the 30 facets of the NEO PI-R (Miller et al., 2001), and that also coincided with the facets that consistently presented significant correlations with different measures of psychopathy in the meta-analysis of O’Boyle et al. (2015), and in four other empirical studies (Lynam et al., 2018). The 11 facets of the NEO PI-R that consistently described psychopathy in the three methods of analysis—content, expert rating, and empirical—were impulsivity (from Neuroticism), excitement-seeking, and low warmth (from Extraversion), low straightforwardness, low altruism, low compliance, low modesty, and low tender-mindedness (from Agreeableness), and low dutifulness, low self-discipline, and low deliberation (from Conscientiousness) (see Supplementary Table 2A).

On the other hand, concerning the most recent conceptions of psychopathy, López Penadés (2010), in a sample of 320 Spanish university students, obtained the correlations between the personality traits of psychopathy as measured by the PPI-R and the facets of the NEO PI-R (see Supplementary Table 3A). Taking into account the facets of the NEO PI-R that showed significant correlations, and of at least a moderate size (≥ | 0.30|) with each of the PPI-R scales in that study, each of these scales was associated with between three and eight facets of the NEO PI-R, except for the Blame Externalization scale which only showed a significant and moderate correlation with the depression facet, although it also showed a significant and almost moderate correlation with the angry hostility (0.29) and trust facets (−0.29).

Regarding the triarchic model of psychopathy, Poy et al. (2014), with a sample of 349 Spanish university students, obtained the correlations between the TriPM scales and the facets of the NEO PI-R (see Supplementary Table 4A). Taking into account the NEO PI-R facets that, both in men and women in that study, showed significant correlations of at least a moderate size (≥ | 0.30|) with each of the TriPM scales, each of these scales was associated with between four and 11 facets of the NEO PI-R.

Finally, concerning the DSM-5’s hybrid model of personality disorders (Sanz-García et al., 2021; Supplementary material) obtained the correlations between the NEO PI-R facets and the PID-5 scales with a sample of 1,052 people from the general Spanish population aged between 16 and 89 years. Taking into account those correlations, the Supplementary Table 5A presents the facets of the NEO PI-R that showed significant correlations of at least a moderate to large size (≥ | 0.40|) with each of the PID-5 scales that measure the personality traits of the antisocial personality disorder with the psychopathy specifier. As can be seen in the Supplementary Table 5A, each of those PID-5 scales is associated with between two and seven facets of the NEO PI-R.

### Definitions of psychopathy based on the Big Five model

The above-mentioned empirical relationships between the NEO PI-R and the four main theoretical perspectives on psychopathy make it possible to propose definitions of psychopathy that allow the comparison and integration of the personality traits that those different perspectives consider as defining psychopathy (that of Hare, operationalized by the PCL-R, that of Lilienfeld, operationalized by the PPI-R, that of the triarchic model, operationalized by the TriPM, and that of the DSM-5 hybrid model, operationalized by the PID-5). In addition, it is possible to propose definitions of psychopathy based on the above-mentioned meta-analyses of the empirical literature on the relationships between the five-factor model and different measures of psychopathy (Supplementary Table 1A), and definitions of psychopathy based on the convergence between those meta-analyses, content analyses mapping the five-factor model facets onto relevant items of a standard psychopathy measure, and analyses based on expert ratings on the five-factor model facets that coincide with the prototype of the psychopath (Lynam et al., 2018; see Supplementary Tables 2A–5A).

In the present study, four criteria were taken into account when establishing those psychopathy definitions. First, as all the theoretical perspectives imply the presence of very high or very low levels of certain personality traits, a very high or very low level in a given personality trait was considered to correspond to a one standard deviation (1 *SD*) above or below, respectively, the average of the general adult population, that is, it corresponded to a T ≥ 60 or ≤ 40 score, respectively.

Second, as the empirical results show that most of the personality traits proposed by the different perspectives on psychopathy relate to various NEO PI-R facets, and some of them share related facets, we selected the facet that appeared consistently in all three methods of analysis performed by Lynam et al. (2018) or that showed a greater correlation and, if two or more traits shared the same related facet, for the trait or traits showing lower correlations, the next related facet was selected (see Supplementary material).

Third, as both the PCL-R cut-off scores and the diagnostic criteria of the hybrid model of personality disorders of the DSM-5 assume that to identify the presence of psychopathy, it is not necessary for all the pathological personality traits to be present, although most of them are, the presence of a minimum number of personality traits was established as a criterion. In the PCL-R, a score of 30 out of 40 is used as a cut-off score to identify psychopathy, a score that has demonstrated good diagnostic and criterion validity (Hare, 2013). As each of the 20 items in the PCL-R can be scored between 0 and 2 and as a score of 2 indicates that the item applies to the person, a score of 30 would mean that 15 of the 20 items apply to the person, that is, 75% of the items. The PCL-R manual also suggests interpreting the total PCL-R score according to different levels, such that a total score of 25 is considered the threshold for a high level of psychopathy (Hare, 2013), and has been used in some research to identify potential, possible, or subclinical psychopathy (e.g., Babiak et al., 2010). A score of 25 would mean that 12 of the 20 items apply to the person, that is, 60% of the items. Following this logic, probable or clinical psychopathy was operationally defined by the presence of 75% of the personality traits proposed by each perspective on psychopathy in its translation into NEO PI-R facets, and potential, possible, or subclinical psychopathy was operationally defined by the presence of 60% of the traits. These operationalizations were not followed in the case of the definition based on the DSM-5 hybrid model, as the DSM-5 already establishes a minimum number of personality traits, in particular, six of the seven traits that define antisocial personality disorder plus the three traits that define the psychopathy specifier (American Psychiatric Association, 2013).

Fourth, as some perspectives of psychopathy assume the existence of two, three, or four dimensions, factors, or subfactors of personality that underlie the pathological personality traits that define psychopathy, it was established as a criterion that at least one of the traits of each of these dimensions, factors, or subfactors should present high or low scores to consider that psychopathy was present. In this sense, Jones and Hare (2016) have argued and stressed the need for any procedure to identify psychopathy to take into account the existence of high scores in each of the PCL-R factors.

### Objectives and hypotheses of this work

Taking into account the aforementioned criteria to establish operational definitions of psychopathy, the primary aim of this work was to examine the prevalence of psychopathy in the general adult population from the currently existing main theoretical perspectives of the construct, using for this purpose the five-factor model as a common language that allows the comparison and integration of the personality traits that these different perspectives consider as defining psychopathy.

Secondarily, the present work was aimed at examining sex differences in psychopathy prevalence in the general population. The results of the meta-analysis of Sanz-García et al. (2021) indicated that psychopathy doubles its prevalence in men compared to women in the general population (7.9% vs. 2.9%). This is coherent with the results of the review of Beryl et al. (2014) that indicated that psychopathy prevalence is also higher in men compared to women in the population of delinquents or incarcerated people. However, the prevalence rates found in the meta-analysis of Sanz-García et al. (2021) also varied significantly depending the type of sample from the general population: higher in samples of workers from organizations and university students than in community samples. In addition, given the small number of studies reviewed in the meta-analysis of Sanz-García et al. (2021), these researchers could not examine the effects of sex while controlling the effect of the type of sample from the general population. Therefore, it may be that sex differences are evident in some types of samples and not in others. For example, Coid et al. (2009) obtained the prevalence of psychopathy in a community sample of the United Kingdom, and the confidence intervals of the prevalence rates in men and women indicated that there were no significant sex differences. In sum, the issue of sex differences in the prevalence of psychopathy in the general population is still an open question, although, based on the scientific literature reviewed by Sanz-García et al. (2021), the hypothesis of the present study was that prevalence rates will be higher in men than in women.

## Materials and methods

### Participants

This study involved 682 adults (390 women and 292 men) aged between 18 and 84 years (mean age = 41.8, *SD* = 14.8) whose responses to the NEO PI-R were used in a previous study on the standardization of this instrument in the general Spanish population (Sanz and García-Vera, 2009). These people were recruited in 2002–2004 using the “snowball” technique by university students of Psychology who invited their relatives and friends to participate in a study on personality and hypertension (*n* = 358) or another one on personality assessment (*n* = 325), although the university students of Psychology themselves did not participate. The sample thus obtained is not random; however, its profile concerning sex and age was very similar to that of the Spanish population in 2004 (see Table 1). More information on the sociodemographic characteristics of the sample appears in Sanz and García-Vera (2009), where it can be seen that the sample was also heterogeneous in the level of studies, marital status, and profession (e.g., 22.1% had primary education as the highest level of education, 30.2% had secondary education, and 45.3% had university education).

**TABLE 1:** Comparison of the distribution by sex and age of the sample of participants with the distribution of the Spanish population (Sanz and García-Vera, 200 9, p. 134).

Age	Sample of participants		Population in Spain (Instituto Nacional de Estadística [INE], 2004)	
	Men (*n* = 292)	Women (*n* = 390)	Men (*N* = 16 243 472)	Women (*N* = 17 262 495)
18 to 29 years	12.2%	15.2%	11.8%	11.3%
30 to 49 years	12.5%	19.8%	18.4%	18.3%
50 years or more	18.2%	22.1%	18.2%	21.9%
Subtotal	42.8%	57.2%	48.5%	51.5%

INE: Instituto Nacional de Estadística (Spanish National Institute of Statistics).

### Instruments and variables

*NEO Personality Inventory-Revised* (NEO PI-R; Costa and McCrae, 1992). The NEO PI-R is a 240-item self-reporting instrument rated on 5-point Likert-type scales, ranging from 0 to 4, designed to evaluate personality based on the Big Five model. The NEO PI-R has five basic scales, each composed of 48 items, which correspond to the basic dimensions of the Big Five, and 30 specific scales of 8 items each (six for each basic scale) that aim to measure the facets or specific personality factors that, according to Costa and McCrae (1992), make up the Big Five. In this study, the Spanish adaptation of the NEO PI-R (Costa and McCrae, 1999) was used. In the present sample of the general Spanish population, the scores of the basic scales of the NEO PI-R obtained excellent reliability coefficients (*r* ≥ 0.85), while the scores of 13 of its specific scales reached good reliability coefficients (0.80 ≤ *r* < 0.85; 1 scale) or appropriate (0.70 ≤ *r* < 0.80; 12 scales), the scores of 12 other specific scales obtained adequate coefficients, although with deficiencies (0.60 ≤ *r* < 0.70), but the remaining five specific scales showed inadequate coefficients (*r* < 0.60). Therefore, the results related to these last five specific scales—the scales of impulsivity, actions, values, tender-mindedness, and competence—should be taken with caution.

*Definitions of psychopathy based on the facets of the NEO PI-R.* Based on the NEO PI-R facets and taking into account the theoretical proposals of psychopathy and criteria described in the introduction as well as the results of the studies on the relationships between the NEO PI-R facets and the measures of psychopathy that are included in the Supplementary material, several operational definitions of psychopathy were established (see also Supplementary Table 6A):

1. *Meta-analytical definition*: based on an equal or higher/lower score (depending on the direction of the relationship with psychopathy) than 1 *SD* above/below mean in at least 10 of the 13 facets (76.9% of the 13 facets) of the NEO PI-R that have shown significant correlations consistent with measures of psychopathy in the meta-analyses (see Supplementary Table 1A), considering subclinical psychopathy if this occurred in at least 8 facets (61.5% of the 13 facets).
2. *Consistent definition among methods*: based on an equal or higher/lower score (depending on the direction of the relationship with psychopathy) than 1 *SD* above/below mean in at least 8 of the 11 facets (72.7% of the 11 facets) of the NEO PI-R that have been shown to consistently describe psychopathy in the following three methods of analysis: FFM translations of psychopathy instruments, expert ratings, and empirical correlations (see Supplementary Table 2A), considering subclinical psychopathy if it occurred in at least 7 facets (63.6% of the 11 facets).
3. *Definition of the PCL-R*: based on an equal or higher/lower score (depending on the direction of the relationship with psychopathy) than 1 *SD* above/below mean in at least 9 of the 12 facets (75% of the 12 facets) of the NEO PI-R describing psychopathy operationalized by the PCL-R (see Supplementary Table 2A), provided that among those 9 was at least one facet describing each of the four subfactors underlying the PCL-R. As some items of the PCL-R were related to several facets of the NEO PI-R, and some of the facets were repeated, the PCL-R’s item concerning absence of remorse or feelings of guilt was not included in the definition because the two NEO PI-R facets that described it already described other PCL-R’s items as well, and hence, 12 NEO PI-R facets were used instead of 13. Therefore, the NEO PI-R facets finally chosen were the following: self-consciousness, modesty, straightforwardness, and altruism to measure PCL-R subfactor 1; tender-mindedness, warmth, and compliance to measure PCL-R subfactor 2; dutifulness, excitement-seeking, self-discipline, and impulsivity, to measure PCL-R subfactor 3; and deliberation to measure PCL-R subfactor 4. With this definition, subclinical psychopathy was considered if the cut-off score was met in at least 7 facets (58.3% of the 12 facets), provided that among those seven was at least one facet that described each of the four subfactors that underlie the PCL-R.
4. *Definition of the PPI-R*: based on an equal or higher/lower score (depending on the direction of the relationship with psychopathy) than 1 *SD* above/below the mean in at least 5 of the 7 facets (71.4% of the 7 facets) of the NEO PI-R describing psychopathy operationalized by the PPI-R (see Supplementary Table 3A), provided that among those 5 was at least one facet describing each of the three factors underlying the PPI-R. As some traits of the PPI-R were related to various facets of the NEO PI-R, and some of the facets were repeated, the definition did not include the PPI-R trait of rebellious non conformity, because the NEO PI-R facet that described it better also described another PPI-R trait, and hence, 7 facets of the NEO PI-R were used instead of 8. Therefore, the following NEO PI-R facets were finally chosen (see Supplementary Table 3A): warmth, excitement-seeking, and anxiety to measure PPI-R factor 1; modesty, trust, and dutifulness to measure PPI-R factor 2; and altruism to measure PPI-R factor 3. With this definition, subclinical psychopathy was considered if the cut-off score was met in at least 4 facets (57.1% of the 7 facets), provided that among those 4, there was at least one facet that described each of the three factors that underlie the PPI-R.
5. *Definition of the TriPM or the triarchic model*. As each of the three psychopathic personality dimensions measured by the TriPM supposedly integrates several personality traits, it was decided to select three NEO PI-R facets for each TriPM dimension (see Supplementary Table 4A): assertiveness, self-consciousness, and vulnerability to measure boldness; altruism, compliance, and straightforwardness to measure meanness; and deliberation, dutifulness, and self-discipline to measure disinhibition. Thus, the psychopathy definition of the triarchic model was based on an equal or higher/lower score (depending on the direction of the relationship with psychopathy) than 1 *SD* above/below the mean in at least 7 of the 9 facets (77.8% of the 9 facets) of the NEO PI-R describing psychopathy operationalized by the TriPM (see Supplementary Table 4A), provided that among those 7 was at least one facet describing each of the three personality dimensions measured by the TriPM (boldness, meanness, and disinhibition), and subclinical psychopathy was considered if the cut-off score was met in at least 5 facets (55.5% of the 9 facets), provided that among those 5 there was at least one facet describing each of those three personality dimensions.
6. *Definition of the PID-5 or DSM-5*: based on an equal or higher/lower score (depending on the direction of the relationship with psychopathy) than 1 *SD* above/below the mean in at least 6 of the 7 facets of the NEO PI-R that describe the personality traits of the antisocial personality disorder according to the DSM-5 and, in addition, in the two NEO PI-R facets that describe the personality traits of the psychopathy specifier according to the DSM-5 (see Supplementary Table 5A). As some traits of the antisocial personality disorder or the psychopathy specifier were related to various NEO PI-R facets, and some of the facets were repeated, the definition did not include the high attention-seeking trait because the two facets that described it also described some traits of the antisocial personality disorder, and hence, two facets of the psychopathy specifier were used instead of three. Therefore, the following NEO PI-R facets were finally chosen: straightforwardness, altruism, modesty, angry hostility, excitement-seeking, deliberation, and dutifulness to measure DSM-5 antisocial personality disorder; and anxiety and warmth to measure DSM-5 psychopathy specifier (see Supplementary Table 5A). With this definition, subclinical psychopathy was considered if the cut-off score was met in at least 4 facets of the antisocial personality disorder (57.1% of the 7 facets) and, in addition, the cut-off score was met in the two facets of the psychopathy specifier.

### Procedure

Participants who collaborated in the personality and hypertension research completed the NEO PI-R as part of a more comprehensive assessment in which they had to fill out other personality questionnaires, with the NEO PI-R being the first. Participants who collaborated in the personality assessment research only completed the NEO PI-R. In both investigations, participants previously signed an informed consent form, and the NEO PI-R was applied individually by the psychology student who had invited the participant to collaborate in one of those two investigations. The training and supervision of the students in the administration of the NEO PI-R were carried out by the last two authors of this study during practical classes or seminars.

### Data analysis

For each of the six definitions of psychopathy, the percentage of adults in the sample of participants who met the criteria for these definitions was calculated. These percentages were also calculated for the men and women in the sample, and the differences according to sex were analyzed by chi-square tests and, in the case of cells with a frequency of less than 5, by Fisher’s exact tests. All statistical analyses were performed using IBM SPSS, version 25.

## Results

Table 2 presents the prevalence rates of psychopathy according to the different definitions of psychopathy. Prevalence rates of clinical psychopathy ranged from 0 to 1%, with a mean of 0.55%, and prevalence rates of subclinical psychopathy ranged from 0.3 to 4.2%, with a mean of 1.65% (see Table 2). The greatest prevalence rates of clinical and subclinical psychopathy were obtained, respectively, with the consistent definition among methods (1%) and the definition based on the triarchic model (4.2%), whereas the lowest prevalence rates were obtained with the definition based on the DSM-5 hybrid model (0% for clinical psychopathy and 0.3% for subclinical psychopathy).

**TABLE 2:** Prevalence of clinical psychopathy and subclinical psychopathy in the sample of participants (*N* = 682) as a function of the different definitions of the psychopathy construct.

Definition of psychopathy	Prevalence (%)	
	Clinical psychopathy	Subclinical psychopathy
Based on meta-analyses	0.4	1.9
Consistent among methods	1.0	1.0
Based on the PCL-R (Hare’s model)	0.4	1.5
Based on the PPI-R (Lilienfeld’s model)	0.9	1.0
Based on the triarchic model	0.6	4.2
Based on the DSM-5 hybrid model	0.0	0.3

As the prevalence rates of clinical psychopathy and subclinical psychopathy found were so small, both prevalence rates were added to analyze the influence of sex. Table 3 presents these combined prevalence rates according to sex and for the different definitions of the two constructs. The prevalence rates of psychopathy were similar in men and women, regardless of the definition of psychopathy used. In fact, chi-square test results revealed no statistically significant difference between men and women in the prevalence of psychopathy (all tests with *p* > 0.05).

**TABLE 3:** Prevalence (%) of clinical psychopathy and subclinical psychopathy in the sample of participants as a function of the different definitions of the psychopathy construct and according to sex (*N* = 292 men and 390 women).

Definition of psychopathy	Prevalence of clinical and subclinical psychopathy	
	Men	Women
Based on meta-analyses	2.4	2.3
Consistent among methods	2.4	1.8
Based on the PCL-R (Hare’s model)	1.0	2.6
Based on the PPI-R (Lilienfeld’s model)	1.4	2.3
Based on the triarchic model	4.5	5.1
Based on the DSM-5 hybrid model	0.3	0.3

## Discussion

Using the five-factor model as a common language that allows the comparison and integration of the personality traits that different perspectives consider as defining psychopathy, the main objective of this work was to examine the prevalence of psychopathy in the general adult population, in particular in Spain, from the main theoretical perspectives on the construct that currently exist—that of Hare, operationalized by the PCL-R, that of Lilienfeld, operationalized by the PPI-R, that of the triarchic model, operationalized by the TriPM, and that of the DSM-5 hybrid model, operationalized by the PID-5—. In addition, this study used definitions of psychopathy based on meta-analyses of the empirical literature on the relationships between the five-factor model and different measures of psychopathy and definitions of psychopathy based on the convergence between those meta-analyses, content analyses mapping the five-factor model facets onto relevant items of a standard psychopathy measure, and analyses based on expert ratings on the five-factor model facets that coincide with the prototype of the psychopath. The results of the present study suggest that the prevalence rates of psychopathy for the different perspectives and definitions were consistently low; specifically, the prevalence of probable or clinical psychopathy could be around 0.55%, and the prevalence of possible, potential, or subclinical psychopathy could be around 1.65%.

This consistent pattern of results was obtained despite the fact that the six psychopathy definitions examined in this study involved 17 facets of the NEO PI-R, and only two facets (altruism and dutifulness) were common to all six definitions and only other five facets (warmth, excitement-seeking, straightforwardness, modesty, and deliberation) were common to five definitions (see Supplementary Table 6A).

The prevalence figures of clinical psychopathy found in the present study are lower than those obtained in Sanz-García et al.’s (2021) meta-analysis on the prevalence of psychopathy in the general population. In this meta-analysis, from 15 studies, an average prevalence of 4.5% was calculated, a prevalence that was reduced to 1.2% when using the PCL-R (or its screening version, the PCL:SV), an instrument that is considered the “gold standard” for the definition and evaluation of psychopathy. However, the meta-analysis also found that the 15 studies were very heterogeneous in terms of the type of sample from the general population, the definitions of psychopathy and the instruments used for its assessment, and that these factors significantly affected the prevalence rates. In this sense, when comparing the results of the present study with the studies of that meta-analysis that examined a more similar sample, that is, a community sample, and that also used a more similar procedure to define psychopathy (a score equal to or greater than 30 in the PCL-R or equal to or greater than 18 in the PCL: SV, which implies the presence of at least 75% of the psychopathic characteristics), the results of these studies (Neumann and Hare, 2008; Coid et al., 2009; Robitaille et al., 2017) are similar to those of the present study. Indeed, in these three studies, prevalence rates of probable or clinical psychopathy of 0.1, 0.2, and 0.9%, respectively, were obtained, from which a weighted average prevalence of 0.27% can be obtained, a figure that is close to the prevalence rate estimated in the present study for probable or clinical psychopathy (0.55%).

In addition, two of these three studies (Neumann and Hare, 2008; Coid et al., 2009) also offered data on the prevalence of subclinical psychopathy (a score equal to or greater than 13 in the PCL:SV, which implies the presence of at least 60%, approximately, of the psychopathic characteristics), from which rates of 0.5 and 1.2%, respectively, can be obtained for this type of psychopathy, figures that are also close to the prevalence rate of subclinical psychopathy obtained in the present study (1.65%).

In summary, the prevalence rates of psychopathy, clinical or subclinical, found in the present study are low and similar to those found in the few previous studies conducted with community samples that used a procedure to define psychopathy similar to the one used in the present study, in the sense that such procedures required the presence of at least 75 and 60% of psychopathic characteristics to identify clinical and subclinical psychopathy, respectively. In fact, among the differences that exist between the studies that have obtained high prevalence rates of psychopathy in the general population (e.g., Hagnell et al., 1994; Love and Holder, 2014; Fritzon et al., 2017) and the studies that, like this one, have obtained low prevalence rates (e.g., Neumann and Hare, 2008; Coid et al., 2009; Robitaille et al., 2017), perhaps the most important has to do with the way clinical and subclinical psychopathy is defined. In this sense, it is important to point out three issues. First, many psychopathy assessment instruments based on models that conceive psychopathy as a maladaptive variant of the normal personality, such as the PPI-R or the LSRP, do not have validated cut-off scores to identify clinical or subclinical psychopathy. Second, most of the studies that have found high prevalence rates of psychopathy have used not validated procedures for defining psychopathy (Hagnell et al., 1994; Love and Holder, 2014; Fritzon et al., 2017), some of those based on the PPI-R or the LSRP (Love and Holder, 2014; Fritzon et al., 2017) and biased to find high prevalence rates (Fritzon et al., 2017). For example, given that most psychopathy assessment instruments based on models that conceive psychopathy as a maladaptive variant of the normal personality provide total scores that are normally distributed in the population, defining clinical psychopathy as 1.5 standard deviations above the mean total score (Fritzon et al., 2017) implies, by definition, obtaining psychopathy prevalence rates of at least 6.7%. Third, in this study, a procedure based on the PCL-R scoring algorithm was used for defining psychopathy. This algorithm underlies the PCL-R cut-off scores that has been validated to identify clinical and subclinical psychopathy (Hare, 2003). In addition, the procedure used in the present study is also partially based on the DSM-5 hybrid model of personality disorders. This model implies the presence of a given number of pathological personality traits from a larger subset of pathological personality traits that define a particular personality disorder. Furthermore, clinical use of the DMS-5 hybrid model involves “the use of formal psychometric instruments designed to measure specific facets and domains of personality” and also involves “comparing individuals’ personality trait levels with population norms” to make “the judgment that a specific trait is elevated (and therefore is present for diagnostic purposes)” (American Psychiatric Association, 2013, p. 774). However, future research will need to clarify whether the psychopathy defining procedure used in this study and the procedures used in the studies by Coid et al. (2009), Neumann and Hare (2008), and Robitaille et al. (2017) are empirically more valid than those used in studies that have found high prevalence rates of psychopathy, since, indeed, definitions and measurements matter.

A question that should be addressed by future research is whether those procedures to define psychopathy—that of the present study and that used by Coid et al. (2009), Neumann and Hare (2008), and Robitaille et al. (2017)—will also obtain low prevalence rates of psychopathy in other types of samples from the general population, such as, for example, workers in some organizations or university students, since, in Sanz-García et al.’s (2021) meta-analysis, the prevalence of psychopathy among workers in some organizations (managers, executives, procurement and supply professionals, advertising workers) was quite high (12.9%), and the prevalence of psychopathy among university students was also high (8.1%).

The fact that, in this study, prevalence rates for the different psychopathy definitions derived from different theoretical perspectives on the construct were consistent reflects the benefits of using a unifying trait-based model, such as the five-factor model, to examine psychopathy. Thus, this study adds to a literature suggesting that the five-factor model provide a parsimonious account of the epidemiologic facts surrounding psychopathy and other personality disorders. For example, Vachon et al. (2013) have found that the use of the five-factor model to obtain measures of psychopathy and antisocial personality disorder predicts the rate of decline for psychopathy over the life span, predicts the differential decline of subfactors of psychopathy, and discriminates the decline of psychopathy from that of antisocial personality disorder. As Hyatt et al. (2020) state: “Using a basic personality framework has substantial benefits, which include parsimony (i.e., the ability to speak about a wide range of personality disorders and related constructs in a common language) and a linkage to a wide, robust, and multifaceted body of research” (p. 74).

The results of the present study also revealed that sex does not seem to have a significant influence on the prevalence of psychopathy in the general population. This finding is not consistent with the results of the meta-analysis of Sanz-García et al. (2021) that indicated that psychopathy doubles its prevalence in men compared to women in the general population (7.9% vs. 2.9%). However, given that, in this study, the absence of sex differences in psychopathy was consistent in the six operational definitions of psychopathy that were analyzed and given that there are no previous studies in this regard carried out with the general Spanish population, it could be speculated whether this absence is a particular characteristic of Spain compared to other countries in which these sex differences have been found in the general population such as, for example, Sweden (Hagnell et al., 1994).

Nonetheless, the explanation for this absence of differences may lie in the small prevalence of psychopathy in the present sample of participants (0.55%, for clinical psychopathy and 1.65% for subclinical psychopathy) and, therefore, that a floor effect has prevented revealing the existence of sex differences. In fact, something similar happened in the study of Coid et al. (2009) with the general population of the United Kingdom, and in which, also with very small prevalences (0.1% for clinical psychopathy and 0.5% for subclinical psychopathy), the confidence intervals of the prevalences in men and women indicated that there were no significant sex differences. In this sense, it is important to note that, in the Swedish study of Hagnell et al. (1994), in which sex differences were found, the prevalence of psychopathy in men was estimated at 8.2% and in women at 3.1%, that is, much higher prevalences than those found in the present study or in the study of Coid et al. (2009).

To rule out the negative influence of this floor effect when examining the influence of sex or other factors in the prevalence of psychopathy in the general adult Spanish population, future research must use larger samples of participants. These future investigations should also try to solve other limitations of this work. In fact, the results and conclusions of this work should be considered in light of these limitations. Among them, an important limitation has to do with the procedure for selecting the sample of participants. This was an incidental sample recruited with the “snowball” technique and, given the inherent limitations of this type of non-probability sampling, its degree of representativeness of the Spanish adult population could be questioned. However, regarding a variable as important as age, the profile of the sample of participants in this study concerning three large age groups (18–29, 30–49, and 50 years and over) was very similar to that found in the Spanish population (see Table 1). Nonetheless, it is obvious that the use of a random sample selection of participants belonging proportionately to different Spanish geographical regions would have greatly improved its representativeness and, therefore, the generalization of the results. In fact, the use of an incidental sample is a common limitation of scientific literature on the prevalence of psychopathy in the general population, since, for example, it affected 12 of the 15 studies (80%) included in Sanz-García et al.’s (2021) meta-analysis on that prevalence.

A second limitation has to do with the evaluation of the psychopathic personality traits and psychopathy from the NEO PI-R rather than using an instrument specifically developed to assess that construct and its defining personality traits such as the PCL-R, the PPI-R, the TriPM, or the PID-5. However, as has been explained in detail in the introduction to this work, the psychopathic personality traits evaluated by these more specific instruments are well reflected by the facets of the NEO PI-R, as evidenced by the content analyses, the analyses based on expert ratings, and the numerous empirical analyses that can be found in the scientific literature, particularly the latter, some of which have been conducted specifically with samples of Spanish participants (Decuyper et al., 2009; Lynam and Miller, 2015; O’Boyle et al., 2015; see tables in the Supplementary material).

Moreover, it could be questioned whether it is possible to evaluate and identify psychopathy simply from the evaluation and presence of extreme personality traits, without taking into account the evaluation and presence of criminal behaviors or the deterioration caused by such traits in social, work, or other important areas of functioning. However, even in samples of the delinquent or prison population, including Spanish samples, several studies have shown that the instruments that evaluate personality traits related to psychopathy but do not include items that evaluate criminal behavior seem to evaluate the same construct of psychopathy (Pedersen et al., 2010; Flórez et al., 2020). In addition, in a recent study, Clark et al. (2019) have demonstrated the strong correlation between measures of personality disorders based on the presence of extreme personality traits and measures based on the presence of problems in the functioning of the personality, such that, although personality traits and personality dysfunction are theoretically distinct constructs, empirically, they are indistinguishable.

Furthermore, the results obtained with some specific instruments for psychopathy such as the PCL-R, the PPI-R, the TriPM, or the PID-5, following a methodology comparable to that used in the present work, are similar to those obtained herein. This similarity has already been discussed before in relation to the few published studies that have identified psychopathy in community samples with procedures to define it that required the presence of at least 75 and 60% of psychopathic characteristics to identify clinical and subclinical psychopathy (Neumann and Hare, 2008; Coid et al., 2009; Robitaille et al., 2017), as assumed by five of the six operational definitions of psychopathy that were examined in the present work. But this similarity can also be seen in the remaining definition, which is based on the hybrid model of personality disorders of the DSM-5.

García et al. (2021) have published on the Internet, as supplementary material, the database they created for their study, which included the PID-5 scores of a sample of 1,052 people from the general Spanish population. From this database, the prevalence of psychopathy has been calculated with the definition of the PID-5 or the DSM-5 with 1 *SD* used in this work, but using the scores on the PDI-5 scales. The prevalence of clinical psychopathy thus obtained in this other sample of the general Spanish population was 0% and that of subclinical psychopathy was also 0%, practically the same as those obtained in the present work with the same definition, but using the NEO PI-R facets: 0% of psychopathy and 0.3% of subclinical psychopathy.

In conclusion, the data of the present study, together with the results of that analysis carried out with the data of García et al. (2021), support the idea that, in Spain, as also seems to occur in the United Kingdom (Coid et al., 2009), USA (Neumann and Hare, 2008), and Canada (Robitaille et al., 2017), the frequency of psychopathy in the general adult population is low and much lower than that estimated in previous works (e.g., Hagnell et al., 1994; Love and Holder, 2014; Fritzon et al., 2017). Therefore, the alarmist claims that are sometimes made, suggesting that, in our daily lives, we are surrounded by psychopathic people who, although not necessarily criminals, can do us a lot of harm psychologically, socially, physically, or economically due to their tendency to perform antisocial or harmful behaviors for others, should be called into question and placed in context.

## Data availability statement

The raw data supporting the conclusions of this article will be made available by the authors upon reasonable request.

## Ethics statement

The studies involving human participants were reviewed and approved by Ethics Committee of the Clinical and Health Psychology Unit of the Complutense University of Madrid. The patients/participants provided their written informed consent to participate in this study.

## Author contributions

Material preparation and data collection were performed by MG-V and JS. Data analyses were performed by JS and AS-G. The first draft of the manuscript was written by AS-G. All authors commented on previous versions of the manuscript, contributed to the study conception and design, read, and approved the final manuscript.

## References

[B1] American Psychiatric Association (2013). *Diagnostic and statistical manual of mental disorders.* Washington DC: American Psychiatric Association.

[B2] Babiak P. Hare R. D. (2019). *Snakes in suits: Understanding and surviving the psychopaths in your office.*

[B3] Babiak P. Neumann C. S. Hare R. D. (2010). Corporate psychopathy: Talking the walk. *Behav. Sci. Law.* 28 174–193. 10.1002/bsl.925 20422644

[B4] Beryl R. Chou S. Völlm B. (2014). A systematic review of psychopathy in women within secure settings. *Personality Indiv. Differ.* 71 185–195. 10.1016/j.paid.2014.07.033

[B5] Clark L. A. Daly E. J. Larew S. Nuzum H. Kingsbury T. Shapiro J. L. (2019). “Personality dysfunction and trait extremity: Conceptually, but not empirically distinct?,” in *Using basic personality research to inform personality pathology*, eds Samuel D. B. Lynam D. R. (Oxford University Press), 122–149. 10.1093/med-psych/9780190227074.003.0006

[B6] Cleckley H. (1976). *The mask of sanity: An attempt to clarify some issues about the so-called psychopathic personality.* St. Louise: C. V. Mosby Company.

[B7] Coid J. Yang M. Ullrich S. Roberts A. Hare R. D. (2009). Prevalence and correlates of psychopathic traits in the household population of great britain. *Int. J. Law Psychiatry* 32 65–73. 10.1016/j.ijlp.2009.01.002 19243821

[B8] Costa P. T. Jr. McCrae R. R. (1992). *Revised NEO Personality Inventory (NEO PI R) and NEO five factor inventory (NEO FFI). Professional manual*. Odessa, FL: Psychological Assessment Resources.

[B9] Costa P. T. Jr. McCrae R. R. (1999). *Inventario NEO reducido de Cinco Factores (NEO-FFI). Manual [Revised NEO Personality Inventory (NEO PI-R). NEO five factor inventory (NEO-FFI)]*. Madrid: TEA Ediciones.

[B10] Crego C. Widiger T. A. (2018). “Antisocial-psychopathic personality disorder,” in *Developmental pathways to disruptive, impulse-control, and conduct disorders*, ed. Martel M. M. (Academic Press), 91–118.

[B11] Decuyper M. De Pauw S. De Fruyt F. De Bolle M. De Clercq B. J. (2009). A meta-analysis of psychopathy-, antisocial PD- and FFM associations. *Eur. J. Personality* 23 531–565. 10.1002/per.729

[B12] Dutton K. (2012). *The wisdom of psychopaths: What saints, spies, and serial killers can teach us about success.* London: Macmillan. 10.1177/147470491301100111 PMC10480796 23446166

[B13] Flórez G. Ferrer V. García L. S. Crespo M. R. Pérez M. Saiz P. A. (2020). Comparison between the psychopathy checklist-revised and the comprehensive assessment of psychopathic personality in a representative sample of spanish prison inmates. *PLoS One* 15:228384. 10.1371/journal.pone.0228384 32023291 PMC7001946

[B14] Fritzon K. Bailey C. Croom S. Brooks N. (2017). “Problem personalities in the workplace: Development of the corporate personality inventory,” in *Psychology and law in Europe: When West meets East*, eds Granhag P. A. Bull R. Shaboltas A. Dozortseva E. (CRC Press), 139–165. 10.1201/9781315317045-13

[B15] Fritzon K. Brooks N. Croom S. (eds) (2020). *Corporate psychopathy: Investigating destructive personalities in the workplace.* Berlin: Springer Nature.

[B16] García L. F. Cuevas L. Lucas I. Aluja A. (2021). Comparing the prediction of dimensional personality disorders (PID-5) after three personality trait models: Five factor, zuckerman, and cloninger models. *Eur. J. Psychol. Assess.* 37 167–177. 10.1027/1015-5759/a000601

[B17] Hagnell O. Ojesjö L. Otterbeck L. Rorsman B. (1994). Prevalence of mental disorders, personality traits and mental complaints in the lundby study. a point prevalence study of the 1957 lundby cohort of 2,612 inhabitants of a geographically defined area who were re-examined in 1972 regardless of domicile. *Scand. J. Soc. Med. Suppl.* 50 1–77. 7846471

[B18] Hare R. D. (1993). *Without conscience: The disturbing world of the psychopaths among us*. New York, NY: Guilford.

[B19] Hare R. D. (2003). The Hare PCL-R: Some issues concerning its use and misuse. *Legal Criminol. Psychol.* 3 99–119. 10.1111/j.2044-8333.1998.tb00353.x

[B20] Hare R. D. (2003). *Hare psychopathy checklist-revised. Technical manual* 2nd Edn. Toronto, ON: Multi-Health Systems.

[B21] Hare R. D. (2013). *PCL-R. escala de evaluación de psicopatía de hare revisada. Manual técnico [PCL-R. hare psychopathy checklist-revised. Technical manual]*. Madrid: TEA Ediciones.

[B22] Hyatt C. S. Crowe M. L. Lynam D. R. Miller J. D. (2020). Components of the triarchic model of psychopathy and the five-factor model domains share largely overlapping nomological networks. *Assessment* 27 72–88. 10.1177/1073191119860903 31304764

[B23] Instituto Nacional de Estadística [INE] (2004). *Censos de población y viviendas 2001. Resultados definitivos [Population and housing census 2001. Definitive results]*. Available online at: http://www.ine.es

[B24] Jones D. N. Hare R. D. (2016). The mismeasure of psychopathy: A commentary on Boddy’s PM-MRV. *J. Bus. Ethics* 138 579-588. 10.1007/s10551-015-2584-6

[B25] Krueger R. F. Derringer J. Markon K. E. Watson D. Skodol A. E. (2012). Initial construction of a maladaptive personality trait model and inventory for DSM-5. *Psychol. Med.* 42 1879–1890. 10.1017/S0033291711002674 22153017 PMC3413381

[B26] Lilienfeld S. O. Widows M. R. (2005). *Psychopathic personality inventory-revised*. Lutz, FL: Psychological Assessment Resources. 10.1037/pas0000032 25436663

[B27] López Penadés R. (2010). *Déficit en el sistema motivacional aversivo en psicópatas subclínicos evaluados mediante el psychopathic personality inventory-revised (PPI-R) [deficit in the aversive motivational system in subclinical psychopaths assessed by the psychopathic personality inventory-revised (PPI-R)]* Ph. D, Thesis.

[B28] Love A. B. Holder M. D. (2014). Psychopathy and subjective well-being. *Personality Indiv. Differ.* 66 112–117. 10.1016/j.paid.2014.03.033

[B29] Lynam D. R. Miller J. D. (2015). Psychopathy from a basic trait perspective: The utility of a five-factor model approach. *J. Personality* 83 611–626. 10.1111/jopy.12132 25204751

[B30] Lynam D. R. Miller J. D. Derefinko K. J. (2018). “Psychopathy and personality: An articulation of the benefits of a trait-based approach,” in *Handbook of psychopathy*, 2nd Edn, ed. Patrick C. J. (The Guilford Press), 259–280.

[B31] McCrae R. R. Costa P. T. Jr. (2003). *Personality in adulthood: A five factor theory perspective*, 2nd Edn. New York: Guilford Press.

[B32] Miller J. D. Lynam D. R. Widiger T. A. Leukefeld C. (2001). Personality disorders as extreme variants of common personality dimensions: Can the five-factor model adequately represent psychopathy? *J. Personality* 69 253–276. 10.1111/1467-6494.00144 11339798

[B33] Neumann C. S. Hare R. D. (2008). Psychopathic traits in a large community sample: Links to violence, alcohol use, and intelligence. *J. Consult. Clin. Psychol.* 76 893–899. 10.1037/0022-006X.76.5.893 18837606

[B34] O’Boyle E. H. Forsyth D. R. Banks G. C. Story P. A. White C. D. (2015). A meta-analytic test of redundancy and relative importance of the dark triad and five-factor model of personality. *J. Personality* 83 644–664. 10.1111/jopy.12126 25168647

[B35] Patrick C. J. (2010). *Operationalizing the triarchic conceptualization of psychopathy: Preliminary description of brief scales for assessment of boldness, meanness, and disinhibition (unpublished manual).* Tallahassee, FL: Department of Psychology, Florida State University.

[B36] Patrick C. J. Fowles D. C. Krueger R. F. (2009). Triarchic conceptualization of psychopathy: Developmental origins of disinhibition, boldness, and meanness. *Dev. Psychopathol.* 21 913–938. 10.1017/S0954579409000492 19583890

[B37] Pedersen L. Kunz C. Rasmussen K. Elsass P. (2010). Psychopathy as a risk factor for violent recidivism: Investigating the psychopathy checklist screening version (PCL:SV) and the comprehensive assessment of psychopathic personality (CAPP) in a forensic psychiatric setting. *Int. J. Forensic Mental Health* 9 308–315. 10.1080/14999013.2010.526681

[B38] Poy R. Segarra P. Esteller À López R. Moltó J. (2014). FFM description of the triarchic conceptualization of psychopathy in men and women. *Psychol. Assess.* 26 69–76. 10.1037/a0034642 24099318

[B39] Robitaille M. Checknita D. Vitaro F. Tremblay R. E. Paris J. Hodgins S. (2017). A prospective, longitudinal, study of men with borderline personality disorder with and without comorbid antisocial personality disorder. *Borderline Personality Dis. Emot. Dysregulat.* 4:25. 10.1186/s40479-017-0076-2 29225887 PMC5719590

[B40] Sanz J. García-Vera M. P. (2009). Nuevos baremos para la adaptación española del inventario de personalidad NEO revisado (NEO PI-R): Fiabilidad y datos normativos en voluntarios de la población general [New norms for the Spanish adaptation of the NEO Personality Inventory-Revised (NEO PI-R): Reliability and norms in volunteers of the general population]. *Clín. Salud* 20 131–144.

[B41] Sanz-García A. Gesteira C. Sanz J. García-Vera M. P. (2021). Prevalence of psychopathy in the general adult population: A systematic review and meta-analysis. *Front. Psychol.* 12:1044. 10.3389/fpsyg.2021.661044 34421717 PMC8374040

[B42] Vachon D. D. Lynam D. R. Widiger T. A. Miller J. D. McCrae R. R. Costa P. T. (2013). Basic traits predict the prevalence of personality disorder across the life span: The example of psychopathy. *Psychol. Sci.* 24 698–705. 10.1177/0956797612460249 23528790

[B43] Widiger T. A. Lynam D. R. (1998). “Psychopathy and the five-factor model of personality,” in *Psychopathy: Antisocial, criminal, and violent behavior*, eds Millon T. Simonsen E. Birket-Smith M. Davis R. D. (The Guilford Press), 171–187.

