# Supplementary material for: Prevalence of psychopathy in a community sample of Spanish adults: Definitions and measurements matter
Source: Front Psychol. 2022 Oct 28;13:997303. doi: 10.3389/fpsyg.2022.997303 (PMC9650134; doi:10.3389/fpsyg.2022.997303)
Supplement: Supplementary file 1 [file Data_Sheet_1.PDF]

ANNEX

Prevalence of Psychopathy in a Community Sample of Spanish Adults: Definitions and Measurements Matter

Ana Sanz García

María Elena Peña Fernández

María Paz García-Vera

Jesús Sanz

Universidad Complutense de Madrid

Spain

Table 1A

Main results of the meta-analyses or joint analyses of several studies on the relationships of psychopathy with NEO PI-R facets

NEO PI-R facets	O'Boyle et al. (2015)<br>Meta-analysis of 76-86 studies<br>*N* = 23 216-25 465	Decuyper et al. (2009)<br>Meta-analysis de 24-25 studies<br>*N* = n. d.	Lynam & Miller (2015)<br>Analysis of 3 studies<br>*N* = 1 482
Neuroticism			
Anxiety	-.03	-.15	**-.20**
**Angry Hostility**	**.37**	**.29**	**.29**
Depression	.10	.05	.04
Self-Consciousness	-.01	-.09	-.11
**Impulsivity**	**.39**	**.24**	**.21**
Vulnerability	.08	.00	.03
Extraversion			
**Warmth**	**-.24**	**-.20**	**-.22**
Gregariousness	.00	.03	.05
Assertiveness	.09	.16	.19
Activity	.06	.07	.04
**Excitement-Seeking**	**.28**	**.31**	**.24**
Positive Emotions	-.17	-.10	-.19
Openness to Experience			
Fantasy	.09	.05	.01
Aesthetics	-.04	-.01	-.05
Feelings	-.07	-.10	-.19
Actions	.09	.09	.14
Ideas	.04	.03	-.02
Values	.06	.00	-.09
Agreeableness			
**Trust**	**-.35**	**-.34**	**-.29**
**Straightforwardness**	**-.56**	**-.61**	**-.58**

NEO PI-R facets	O'Boyle et al. (2015)	Decuyper et al. (2009)	Lynam & Miller (2015)
	Meta-analysis of 76-86 studies *N* = 23 216-25 465	Meta-analysis de 24-25 studies *N* = n. d.	Analysis of 3 studies *N* = 1 482
**Altruism**	**-.40**	**-.41**	**-.47**
**Compliance**	**-.47**	**-.48**	**-.42**
**Modesty**	**-.25**	**-.32**	**-.38**
**Tender-Mindedness**	**-.36**	**-.31**	**-.33**
Conscientiousness			
Competence	**-.23**	-.17	**-.29**
Order	**-.25**	-.17	**-.20**
**Dutifulness**	**-.41**	**-.32**	**-.35**
Achievement Striving	**-.26**	-.11	-.17
**Self-Discipline**	**-.31**	**-.22**	**-.24**
**Deliberation**	**-.46**	**-.38**	**-.46**

Note. Statistically significant correlations of at least $p < .05$ are presented in boldface. The NEO PI-R facets that present significant correlations with psychopathy in the three works are presented in bold. n. d. = no data in the published article.

Table 2A

Correspondence between the personality traits of psychopathy measured by PCL-R and the NEO PI-R facets based on the content analysis of Widiger and Lynam (1998) (adapted from Derefinko & Lynam, 2013, p. 105)

PCL-R Subfactors	NEO PI-R Facets
Subfactor 1: Interpersonal	
1. Glibness/superficial charm	Self-Consciousness (-)
2. Grandiose sense of self-worth	**Modesty (-)**
4. Pathological lying	**Straightforwardness (-)**
5. Conning/manipulative	**Straightforwardness (-)**
	**Altruism (-)**
	**Tender-Mindedness (-)**
Subfactor 2: Affective	
6. Lack of remorse or guilt	**Tender-Mindedness (-)**
7. Emotional shallow	**Warmth (-)**
	Positive Emotions (-)
	**Altruism (-)**
	**Tender-Mindedness (-)**
8. Callous/lack of empathy	**Modesty (-)**
	**Tender-Mindedness (-)**
16. Failure to accept responsibility for own actions	**Trust (-)**
	**Compliance (-)**
	**Tender-Mindedness (-)**
	**Dutifulness (-)**
Subfactor 3: Lifestyle	
3. Need of stimulation/proneness to boredom	**Excitement-Seeking (+)**
	**Self-Discipline (-)**
13. Lack of realistic, long-term goals	Achievement Striving (-)
	**Self-Discipline (-)**
	**Deliberation (-)**
14. Impulsivity	**Impulsivity (+)**
	**Deliberation (-)**

PCL-R Subfactors	NEO PI-R Facets
15. Irresponsibility	**Dutifulness (-)**
Subfactor 4: Antisocial	
10. Poor behavioral control	Angry Hostility (+)
	**Deliberation (-)**
	**Compliance (-)**

Note. The items of the PCL-R that measure behaviors rather than personality traits are not included: items that measure a parasitic lifestyle (Item 9), early behavioral problems (Item 12), juvenile delinquency (Item 18), revocation of conditional release (Item 19), and criminal versatility (Item 20). The NEO PI-R facets that, according to the results of Lynam et al. (2018), coincide with the prototype of the psychopath according to expert ratings and that also correlate significantly with different measures of psychopathy according to the results of a meta-analysis and different empirical studies are presented in bold.

Table 3A

Correlation between the personality traits of psychopathy measured by the PPI-R and the NEO PI-R facets according to the results of López Penadés (2010)

PPI-R Traits	NEO PI-R Facets
Factor 1: Fearless Dominance	
Social Influence	Activity
	**Warmth**
	Gregariousness
	Assertiveness
	Positive Emotions
	Self-Consciousness (-)
	Feelings
	**Modesty (-)**
Fearlessness	**Excitement-Seeking**
	Activity
	Feelings
Stress Immunity	Anxiety (-)
	Depression (-)
	Self-Consciousness (-)
	Vulnerability (-)
Factor 2: Self-Centered Impulsivity	
Machiavellian egocentricity	**Modesty (-)**
	**Altruism (-)**
	Trust (-)
Rebellious Nonconformity	**Excitement-Seeking**
	Feelings
Blame Externalization	Trust (-)[a]

PPI-R Traits	NEO PI-R Facets
Carefree Nonplanfulness	Achievement Striving (-)
	**Dutifulness (-)**
	**Deliberation (-)**
	**Self-Discipline (-)**
	Competence (-)
	Order (-)
Factor 3: Coldheartedness	**Altruism (-)**
	**Tender-Mindedness (-)**
	**Modesty (-)**
	Anxiety (-)
	Depression (-)

Note. The NEO PI-R facets that had significant correlations of at least a moderate size (≥ |.30|) with each of the scales of the PPI-R in the study of López Penadés (2010) with a sample of 320 Spanish university students are shown. The NEO PI-R facets are presented in bold that, according to the results of Lynam et al. (2018), coincide with the prototype of the psychopath according to expert ratings and with the results of content analysis and that also correlate significantly with different measures of psychopathy according to the results of a meta-analysis and different empirical studies. [a]In the case of the Blame Externalization scale, the only NEO PI-R facet that, in the study of López Penadés (2010), showed a significant correlation of moderate size with that scale was the depression facet, with a positive correlation of .30; however, the second facet of the NEO PI-R with a greater correlation with the scale of Blame Externalization was the trust facet, with a significant and negative correlation of almost moderate size (-.29), and, as its content in the negative sense is more theoretically related to the scale of Blame Externalization than the content of the facet of depression in a positive sense, we decided to replace this facet with the trust facet.

Table 4A

Correlation between the personality traits of psychopathy measured by the TriPM and the NEO PI-R facets according to the results of Poy et al. (2014)

TriPM Traits	NEO PI-R Facets
Boldness	Assertiveness
	Self-Consciousness (-)
	Vulnerability (-)
	Depression (-)
	Anxiety (-)
	Positive Emotions
	Activity
	Trust (-)
	Actions
	**Modesty (-)**
	**Warmth**
Meanness	**Altruism (-)**
	**Compliance (-)**
	**Dutifulness (-)**
	**Straightforwardness (-)**
Disinhibition	**Deliberation (-)**
	**Dutifulness (-)**
	Competence (-)
	**Self-Disciplines (-)**
	**Impulsivity**
	Angry Hostility
	Depression
	Trust (-)
	**Compliance (-)**
	**Straightforwardness (-)**

Note. The NEO PI-R facets are shown that presented, both in men and women, significant correlations of at least a moderate size (≥ |. 30|) with each of the scales of the TriPM in the study of Poy et al. (2014) with a sample of Spanish university students, 253

women and 96 men. In bold are presented the NEO PI-R facets that, according to the results of Lynam et al. (2018), coincide with the prototype according to expert ratings and with the results of content analysis and that also correlate significantly with different measures of psychopathy according to the results of a meta-analysis and different empirical studies.

Table 5A

Correlation between the personality traits of psychopathy measured by the PID-5 and the NEO PI-R facets according to the results of García et al. (2021, Supplementary material)

PID-5 Traits	NEO PI-R Facets
Antisocial Personality Disorder	
Manipulativeness	**Straightforwardness (-)**
	**Modesty (-)**
Callousness	**Altruism (-)**
	**Straightforwardness (-)**
	**Warmth (-)**
	**Compliance (-)**
	Trust (-)
Deceitfulness	**Straightforwardness (-)**
	**Modesty (-)**
	**Dutifulness (-)**
	**Altruism (-)**
Hostility	Angry Hostility
	**Compliance (-)**
Risk-Taking	**Excitement-Seeking**
	**Deliberation (-)**
Impulsivity	**Deliberation (-)**
	**Impulsivity**
	Competence (-)
	Angry Hostility
Irresponsibility	**Dutifulness (-)**
	**Self-Discipline (-)**
	Competence (-)
	**Deliberation (-)**
	**Impulsivity**
	Order (-)
	Achievement Striving (-)
Psychopathy Specifier	

PID-5 Traits	NEO PI-R Facets
Low Anxiousness	Anxiety (-)
	Depression (-)
	Vulnerability (-)
	Self-Consciousness (-)
	Hostility (-)
Low Withdrawal	**Warmth**
	Gregariousness
	Positive Emotions
Attention Seeking	**Modesty (-)**
	**Straightforwardness (-)**

Note. The NEO PI-R facets are shown that presented significant correlations and of at least a moderate to large size (≥ |. 40|) with each of the PID-5 scales in the study of García et al. (2021, Supplementary material) with a sample of 1052 people from the general Spanish population aged between 16 and 89 years. In bold are presented the NEO PI-R facets that, according to the results of Lynam et al. (2018), coincide with the prototype according to expert ratings and with the results of content analysis and that also correlate significantly with different measures of psychopathy according to the results of a meta-analysis and different empirical studies.

Table 6A

Summary of the NEO PI-R facets that make up each definition of psychopathy and, where appropriate, correspondence of the NEO PI-R facets with the personality traits of the different models of psychopathy

Facets of NEO PI-R	Definition of psychopathy					
	Meta-analyses	Consistent among methods	Hare's model	Lilienfeld's model	Triarchic model	DSM-5 model
Anxiety				Stress immunity		Low anxiousness
Angry Hostility	X					Hostility
Self-Consciousness			Glibness/superficial charm		Boldness	
Impulsivity	X	X	Impulsivity			
Vulnerability					Boldness	
Warmth	X	X	Emotional shallow	Social influence		Low withdrawal
Assertiveness					Boldness	
Excitement-Seeking	X	X	Need of stimulation/proneness to boredom	Fearlessness<br>Impulsive nonconformity		Risk-taking
Trust	X	X	Failure to accept responsibility for own actions	Blame externalization		
Straightforwardness	X	X	Pathological lying		Meanness	Manipulativeness
Altruism	X	X	Conning/manipulative	Coldheartedness	Meanness	Callousness

Facets of NEO PI-R	Definition of psychopathy					
	Meta-analyses	Consistent among methods	Hare's model	Lilienfeld's model	Triarchic model	DSM-5 model
Compliance	X	X	Failure to accept responsibility for own actions		Meanness	
Modesty	X	X	Grandiose sense of self-worth	Machiavellian egocentricity		Deceitfulness<br>Attention seeking
Tender-Mindedness	X	X	Lack of remorse or guilt<br>Callous/lack of empathy			
Dutifulness	X	X	Irresponsibility	Carefree nonplanfulness	Disinhibition	Irresponsibility
Self-Discipline	X	X	Lack of realistic, long-term goals		Disinhibition	
Deliberation	X	X	Poor behavioral control		Disinhibition	Impulsivity
No. of NEO PI-R facets:	13	12	13	7	9	9

References

Decuyper, M., De Pauw, S., De Fruyt, F., De Bolle, M., & De Clercq, B. J. (2009). A meta-analysis of psychopathy-, antisocial PD- and FFM associations. *European Journal of Personality, 23*(7), 531-565. http://dx.doi.org/10.1002/per.729

Derefinko, K., & Lynam, D. R. (2013). Psychopathy from the perspective of the five-factor model of personality. In T. A. Widiger & P. T. Costa, Jr. (Eds.), *Personality disorders and the five-factor model of personality* (pp. 103-117). American Psychological Association. https://doi.org/10.1037/13939-007

García, L. F., Cuevas, L., Lucas, I., & Aluja, A. (2021). Comparing the prediction of dimensional personality disorders (PID-5) after three personality trait models: Five factor, Zuckerman, and Cloninger models. *European Journal of Psychological Assessment, 37*(3), 167-177. http://dx.doi.org/10.1027/1015-5759/a000601

López Penadés, R. (2010). *Déficit en el sistema motivacional aversivo en psicópatas subclínicos evaluados mediante el Psychopathic Personality Inventory-Revised (PPI-R)* [Deficit in the aversive motivational system in subclinical psychopaths assessed by the Psychopathic Personality Inventory-Revised (PPI-R)] (Tesis doctoral, Universitat Jaume I). http://hdl.handle.net/10803/384630

Lynam, D. R., & Miller, J. D. (2015). Psychopathy from a basic trait perspective: The utility of a five-factor model approach. *Journal of Personality, 83*(6), 611-626. https://doi.org/10.1111/jopy.12132

Lynam, D. R., Miller, J. D., & Derefinko, K. J. (2018). Psychopathy and personality: An articulation of the benefits of a trait-based approach. In C. J. Patrick (Ed.), *Handbook of psychopathy* (2nd ed., pp. 259-280). The Guilford Press.

O'Boyle, E. H., Forsyth, D. R., Banks, G. C., Story, P. A., & White, C. D. (2015). A meta-analytic test of redundancy and relative importance of the dark triad and five-factor

model of personality. *Journal of Personality, 83*(6), 644-664. http://dx.doi.org/10.1111/jopy.12126

Poy, R., Segarra, P., Esteller, À., López, R., & Moltó, J. (2014). FFM description of the triarchic conceptualization of psychopathy in men and women. *Psychological Assessment, 26*(1), 69-76. http://dx.doi.org/10.1037/a0034642

Widiger, T. A., & Lynam, D. R. (1998). Psychopathy and the five-factor model of personality. In T. Millon, E. Simonsen, M. Birket-Smith & R. D. Davis (Eds.), *Psychopathy: Antisocial, criminal, and violent behavior* (pp. 171-187). The Guilford Press.
